# Kidney tissue elastography and interstitial fibrosis observed in kidney biopsy

**DOI:** 10.1080/0886022X.2022.2035763

**Published:** 2022-02-15

**Authors:** Mehmet Sami Islamoglu, Sibel Gulcicek, Nurhan Seyahi

**Affiliations:** a Department of Internal Medicine, Faculty of Medicine, Biruni University, Istanbul, Turkey; b Department of Nephrology, Istanbul Training and Research Hospital, Istanbul, Turkey; c Division of Nephrology, Department of Internal Medicine, Cerrahpasa Medical Faculty, Istanbul University, Istanbul, Turkey

**Keywords:** Elastography interstitial fibrosis, kidney kidney biopsy

## Abstract

**Introduction:**

Kidney interstitial fibrosis is an important risk factor for the progression of chronic kidney disease. Kidney elastography is a noninvasive imaging modality that might be used to assess tissue fibrosis. In this study, we aimed to investigate the relationship between tissue stiffness detected in kidney elastography and interstitial fibrosis observed in kidney biopsy.

**Materials and methods:**

Patients who were hospitalized in a tertiary care university hospital with a kidney biopsy indication were included in this study. In all patients, the transverse and sagittal elastography measurements were made using a sonoelastography device before the biopsy. The total histological score was calculated.

**Results:**

Fifty-seven native kidney patients with proteinuria were included in the study. Patients were divided into two groups according to the presence (*n* = 6) and absence of fibrosis (*n* = 51) as detected by kidney biopsy. A significant correlation was found between the presence of fibrosis detected by biopsy and elastography outcomes (*p* = .046, *r* = .192). A significant correlation was found between the urea and creatinine levels and transverse elastography measurements (*p* = .036, *r* = .240). No correlation was observed between the transverse elastography measurements and total histological score consisting of glomerular, vascular, and tubular scores (*r* = .006, *p* = .967)

**Conclusion:**

The findings of our study suggest a significant relationship between the elastography measurements and interstitial fibrosis. Because of the high negative predictive value (91%), we suggest that elastography should mainly be used as an exclusion test for the presence of fibrosis. We also believe that elastography may be useful to evaluate the fibrosis status in kidney diseases.

## Introduction

Chronic kidney disease is becoming a major public health problem worldwide [1]. While the number of patients requiring kidney replacement therapy in the USA was 340,000 in 1999, it increased to 651,000 in 2010 [2]. Chronic kidney disease, which progresses crescively and irreversibly, is associated with high cardiovascular risk and has a prevalence of 10–13% in the general population [3]. Glomerulosclerosis and kidney interstitial fibrosis, which cause the progression of chronic kidney disease, are targeted during treatment. Chronic tubulointerstitial disease, which leads to chronic kidney disease, causes glomerular, vascular, and tubulointerstitial damage, eventually leads to interstitial scarring, fibrosis, and tubular atrophy. It is critical to understand the link between the ‘supersonic shear imaging’ (SSI) technique, a novel and promising approach for assessing the elasticity of numerous organs, including the liver, and pathologically identifiable interstitial fibrosis and overall histological score in chronic kidney disease. Histopathological diagnosis with kidney biopsy gives important information for the prognosis and management of chronic kidney disease, while kidney biopsy has complications such as bleeding, fistula, and rarely death [4,5]. Proteinuria and eGFR (estimated glomerular filtration rate) are important and noninvasive biomarkers of chronic kidney disease, however, studies for developing early and more sensitive biomarkers are ongoing [6]. In addition to biomarkers, observation of the course of the disease with advanced ultrasound or magnetic resonance-based or molecular imaging techniques is also promising [7]. In elastography, which is a novel ultrasound-based method, tissue stiffness or elasticity is measured using ultrasound [8]. Tissue stiffness is measured using Young’s modulus and expressed in kilopascals or pascals [9]. Young’s elastic modulus, as evaluated by noninvasive elastography, correlates with the degree of interstitial fibrosis [10]. Among various elastography methods, the SSI method is the most appropriate method to evaluate the stiffness of natural kidneys [8]. This technique is a simultaneous and quantitative method that has been successfully applied to curved surfaces for mapping tissue elasticity [9].

In our study, we investigated the relationship between the interstitial fibrosis detected in biopsy and the total histological score obtained by the scoring of the histopathological findings and the kidney tissue stiffness determined by the SSI method.

## Materials and methods

Fifty-seven native kidney patients with proteinuria who were hospitalized or examined at the outpatient clinic of a tertiary care university hospital with a kidney biopsy indication were included in the study. The inclusion criteria were: patients who were over 18 years of age, in whom kidney biopsy was indicated, and those who showed cooperation to have elastography examination. Patients who did not give informed consent to participate in the study, underwent kidney transplantation, had severe heart failure and urinary obstruction, were on dialysis, had suffered from neurological illnesses such as delirium dementia, and patients with data loss were excluded.

The patients’ demographic information, diagnoses, kidney biopsy indications, laboratory results (urea, creatinine, glomerular filtration rate [GFR], proteinuria in 24-h urine sample), and pathology information were obtained from the patient files. Ethical approval for the study was obtained from the Ethics Committee of Cerrahpaşa Faculty of Medicine at Istanbul University (No: 12452).

**Figure 1.**
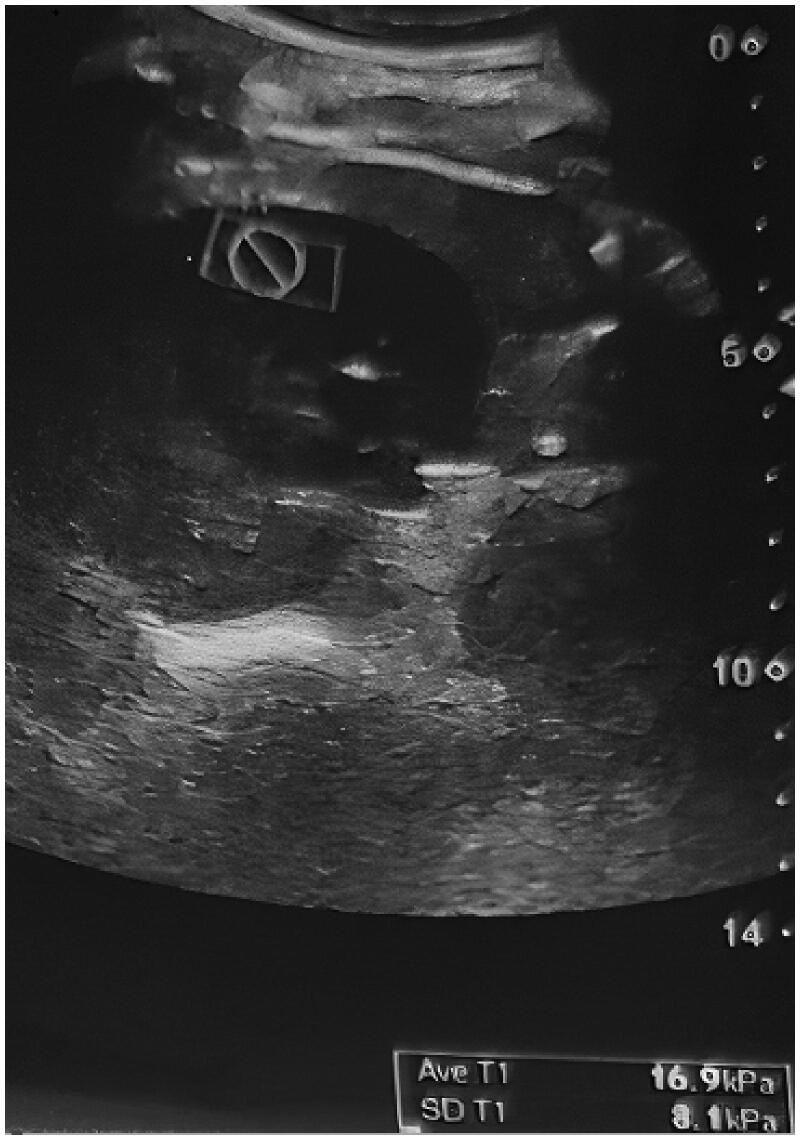
Sagittal supersonic shear imaging elastography image.

**Figure 2.**
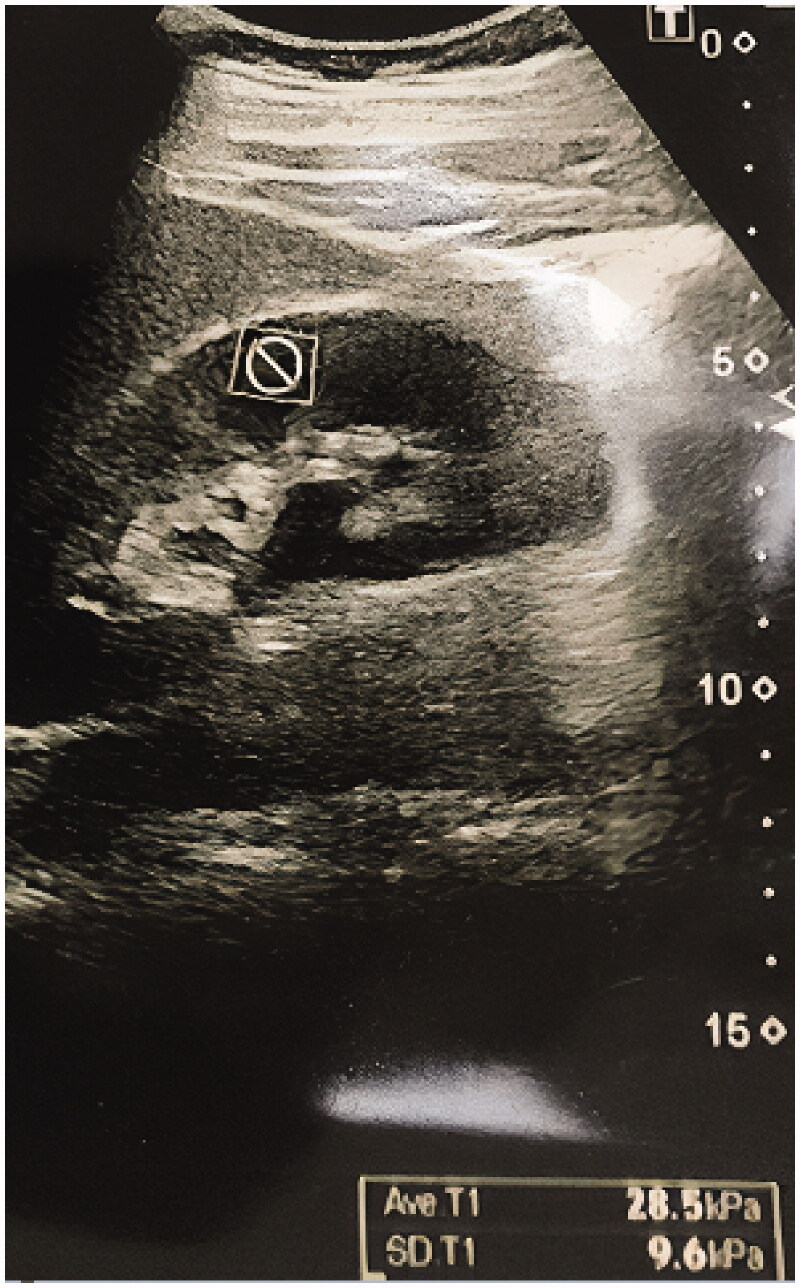
Transverse supersonic shear imaging elastography image.

Supersonic shear imaging elastography was performed using a sonoelastography device (Aixplorer; SuperSonic Imagine, Aix-en-Provence, France) and 4 MHz transducers before biopsy in all patients. The patients were placed on a stretcher in the supine position. The left native kidney was visualized using B-mode ultrasound imaging. The color scale formed as a result of the signals obtained from the inferior pole and the cortex of the kidney using SSI elastography was evaluated in kilopascals (kPa) using the Tsukuba scoring system. The elastography measurements were made both in the sagittal and transverse sections. Sagittal and transverse elastography images are shown in Figures 1 and 2. The measurements of cortical elasticity were made by a single radiologist.

Biopsies were obtained from the same left kidney as the ultrasound measurement has taken place. Biopsies of the patients were evaluated at the Department of Pathology. The routine hematoxylin & eosin, Masson’s trichrome, and PAS stainings were used to detect interstitial fibrosis. The glomerular score, tubulointerstitial score, and vascular score were also calculated to evaluate the presence of interstitial fibrosis. The kidney histological total score was calculated by recording the glomerular score (3–12 points), tubulointerstitial score (3–9 points), and vascular score (3–6 points) according to the percentage of involvement, as suggested by Hu et al. [11]. Segmental and global glomerulosclerosis percentages were evaluated with the glomerular score, tubular atrophy, tubular cell infiltration, and interstitial fibrosis percentages with the tubulointerstitial score, and the arterial hyalinosis percentage with the vascular score. A total score of ≤9 was considered as a ‘mild’ impairment, a score of 10–18 as a ‘moderate’ impairment, and a score of 19 and above as a ‘severe’ impairment. The correlation between the total histological score and elastography measurements was also evaluated. Apart from the total histological score, the patients were also evaluated based on the presence of interstitial fibrosis. The patients were separated into two groups: those with interstitial fibrosis (*n* = 6) and those without it (*n* = 51). Patients with interstitial fibrosis in more than 25% of the pathological sample were assigned to the fibrosis group, whereas patients with interstitial fibrosis in 25% or lesser section of the sample or no fibrosis on pathological examination were assigned to the non-fibrosis group. The presence of interstitial fibrosis in the biopsies was compared to the elastography measurements.

### Statistical analysis

The SPSS for Windows v.17.0 software was used in all statistical analyses. The data are expressed as mean ± SD. The distribution of the variables and the relationship between them were evaluated with the independent samples t-test. The intergroup elastography measurements, age, creatinine, and global sclerosis were analyzed with one-way analysis of variance (ANOVA). The chi-square test was used for the analysis of the nominal data, while Pearson’s correlation test was used to evaluate the correlation between the numerical variables. The statistical significance level was set at *p* < .05.

## Results

The demographic characteristics, clinical, and laboratory findings of the patients are given in Table 1. The patients were middle-aged with an average age of 41.4 years and with a slight predominance of the male gender. In the evaluation of the creatinine and GFR levels, we observed that the patients did not develop end-stage renal disease but had overt proteinuria. Table 2 shows the comparison of the patients based on the presence of fibrosis. The elastography measurements in the transverse sections revealed a kidney stiffness of 12.83 ± 9.80 kPa in the fibrosis group and 7.76 ± 5.16 kPa in the non-fibrosis group (*n* = 51), exhibiting a significant difference between the groups (*p* = .046). The whole cohort had a mean total histological score of 4.49 ± 3.3, a glomerular score of 2.64 ± 1.85, a tubular score of 1.45 ± 1.48, and a vascular score of 0.22 ± 0.25. The total histological score was found to be positively correlated with age (*r* = 0.428, *p* = .001), creatinine level (*r* = 0.759, *p* < .001), global sclerosis percentage (*r* = 0.874, *p* < .001), and proteinuria (*r* = 0.316, *p* = .017), whereas it showed a negative correlation with GFR (*r* = –0.537, *p* < .001). No correlation was observed between the transverse elastography measurements and the total histological score (*r* = 0.006, *p* = .967) (Table 3).

**Table 1.** The demographic and laboratory data of the patients included in the study.

	Patients (*n* = 57) Mean ± SD
Male gender (%)	54.4
Age	41.46 ± 13.53
Urea (mg/dL)	44.38 ± 27.15
Creatinine (mg/dL)	1.26 ± 1.15
GFR (ml/min/1.73m^2^)	92.42 ± 51.09
Proteinuria (mg)	3,144.1 ± 3,191.7

GFR: glomerular filtration rate.

**Table 2.** Comparison of the demographic, clinical, and laboratory data of the groups according to the presence of biopsy-derived fibrosis.

	With fibrosis (*n* = 6) Mean ± SD	Without fibrosis (*n* = 51) Mean ± SD	*p*
Age	42.33 ± 8.73	41.36 ± 14.05	NS
Urea (mg/dL)	46.50 ± 32.00	44.12 ± 26.88	NS
Creatinine (mg/dL)	1.61 ± 1.82	1.22 ± 1.06	NS
GFR (mL/min/1.73m^2^)	87.33 ± 40.17	93.04 ± 52.55	NS
Sagittal elastography (kPa)	12.00 ± 8.29	9.41 ± 6.18	NS
Transverse elastography (kPa)	12.83 ± 9.80	7.76 ± 5.16	**.046**
Proteinuria (mg)	3,456.00 ± 3,044.46	3,166.04 ± 3,245.61	NS
Global sclerosis score	1.00 ± 0.63	1.00 ± 0.75	NS
Segmental sclerosis score	0.83 ± 0.75	0.72 ± 0.60	NS

GFR: glomerular filtration rate, NS: not significant, SD: standard deviation.

Significant *p* values are written in bold.

**Table 3.** The correlation between the clinical data and total histological score and elastography measurements in the transverse section.

	Total histological score		Transverse elastography	
	r	*p*	r	*p*
Age	0.428	**.001**	0.140	.299
Creatinine	0.759	**<.001**	0.012	.927
GFR	−0.537	**<.001**	0.192	.152
Global sclerosis percentage	0.874	**<.001**	−0.057	.673
Total histological score	1		0.006	.967
Presence of fibrosis	0.206	.125	0.053	.695
Transverse elastography	0.006	.967	1	
Proteinuria	0.316	**.017**	0.198	.140

GFR: glomerular filtration rate.

Significant *p* values are written in bold.

We also performed a ROC curve analysis and calculated the threshold value for the diagnosis of fibrosis as 6.50 kPa. Accordingly, we calculated the relevant values for the sensitivity (50%), specificity (62%), positive predictive value (13%), and negative predictive value (91%).

## Discussion

The prevalence of end-stage chronic kidney disease is increasing all around the world [3]. Although kidney length is a simple parameter that shows kidney functions, it has been recently discovered that cortical thickness measurement has a stronger relationship with kidney functions [12,13]. Hoi et al. detected a strong correlation between cortical thickness and kidney function [14]. The fibrotic process, which progresses due to the formation of interstitial fibrosis or glomerulosclerosis, accelerates the progression to chronic kidney disease. Despite its invasive nature and complications, kidney biopsy is still the gold standard in detecting fibrosis, since recently studied ideal noninvasive markers, such as elastography, is limited [15]. In their study, Moon et al. [16] evaluated the tissue stiffness by elastography, and the kidney dimensions, resistive index, and echogenicity by ultrasonography. The authors divided the 38 kidneys of 19 rabbits into three subgroups and found a correlation between the degree of fibrosis and renal tissue stiffness, however, they observed no difference among the groups in terms of resistive index and other sonographic parameters. Unlike this study, Dalkiran et al. recommended the use of the resistive index in the differentiation of atrophy and hypoplasia in their study of 63 patients [17]. In another study of 202 patients with chronic renal failure, Hanamura et al. showed that the resistive index increased in cases of glomerulosclerosis, arteriolosclerosis, and tubulointerstitial damage [18]. Although the SSI elastography method is the most ideal elastographic method, different results have been reported in different studies. Derieppe et al. investigated the relationship between the cortical tissue elasticity determined by the SSI elastography method and the histopathological development of fibrosis in the kidney in their experimental study on a mouse model and detected a relationship between impaired kidney function and increased tissue elasticity [19]. Contrary to this finding, Desvignes et al. found no relationship between elasticity and fibrosis in their study of 95 children and adolescents, 31 of whom were transplant recipients [20].

In their study of 177 patients, Jenkins et al. found a significant relationship between the global and segmental sclerosis percentages and interstitial fibrosis, and between the global and segmental sclerosis percentages and serum creatinine level [21]. Turgutalp et al. found a correlation between interstitial fibrosis and elastography in a study involving 30 patients with IgA nephropathy and 32 healthy controls [10]. Jenkins et al.’s findings are similar to those of our study [21]. In our study, we observed that the elastography transverse measurement value was significantly different in the group with fibrosis than the group without fibrosis. In a study of 76 patients with chronic kidney disease, Guanghe et al. measured tissue elasticity quantitatively with the ARFI (acoustic radiation force impulse) imaging technique and found a significant difference between the groups with and without fibrosis [22]. Our study comprised of patients with proteinuria and a kidney biopsy justification with a mean GFR greater than 60, which differs from previous studies owing to the lack of patients with end-stage chronic kidney disease and extensive fibrosis. In another study by Iyama et al., the authors found no correlation between kidney elasticity and interstitial fibrosis and between kidney elasticity and glomerular sclerosis [23]. Similar to our study, Hu et al. found a correlation between elastography measurements and histological score, and between elastography measurements and creatinine level in their study on 162 chronic kidney disease patients and 32 healthy patients [11]. In another study on 75 patients with chronic kidney disease, Leong et al. found that a kidney tissue stiffness above 5.81% kPa was associated with glomerular sclerosis, tubular atrophy, and interstitial fibrosis [24].

Because of the high negative predictive value, we suggest that elastography should mainly be used as an exclusion test for the presence of fibrosis. However, we want to point out that because of the low number of patients with fibrosis our results should be confirmed in larger groups. Indeed, the most important limitation of our study was the small number of patients in the fibrosis group, since patients with proteinuria with new onset of chronic kidney disease or developing acute kidney disease rather than the end-stage chronic kidney disease group were included in the study. Despite these limitations, a significant relationship was observed between elastography measurements and fibrosis. If we included the patients with end-stage chronic kidney disease in whom fibrosis is more prevalent, the significant relationship could have been established more strongly. Further studies with more chronic kidney disease patients are needed.

In conclusion, we found a significant relationship between the kidney shear wave elastography measurements and the fibrosis detected in kidney biopsy. The prevalence of chronic kidney disease and consequent end-stage chronic kidney disease is increasing gradually and has become an important public health problem due to its increasing prevalence and morbidity. Since fibrosis is an important risk factor for the progression of chronic kidney disease, we recommend kidney elastography as a key noninvasive method to evaluate fibrosis.

## Author contributions

All authors contributed significantly to the design of the study and acquisition of the data.
